# Kinetic Landscape of Single Virus-like Particles Highlights the Efficacy of SARS-CoV-2 Internalization

**DOI:** 10.3390/v16081341

**Published:** 2024-08-22

**Authors:** Aleksandar Atemin, Aneliya Ivanova, Wiley Peppel, Rumen Stamatov, Rodrigo Gallegos, Haley Durden, Sonya Uzunova, Michael D. Vershinin, Saveez Saffarian, Stoyno S. Stoynov

**Affiliations:** 1 Institute of Molecular Biology, Bulgarian Academy of Sciences, 21, G. Bontchev Str., 1113 Sofia, Bulgaria; atemin@bio21.bas.bg (A.A.); anivanova@bio21.bas.bg (A.I.); stamatov@bio21.bas.bg (R.S.); sonyauzunova84@gmail.com (S.U.); 2 Department of Physics and Astronomy, University of Utah, Salt Lake City, UT 84112, USA; wileypeppel@gmail.com (W.P.); u0966311@utah.edu (R.G.); hdurden84@gmail.com (H.D.); 3 Center for Cell and Genome Science, University of Utah, Salt Lake City, UT 84112, USA; 4 Department of Biology, University of Utah, Salt Lake City, UT 84112, USA

**Keywords:** SARS-CoV-2, SARS-CoV-2 dynamics, virus internalization, Omicron, nucleocapsid release, virus-like particles, dynamin, single-particle tracking

## Abstract

The efficiency of virus internalization into target cells is a major determinant of infectivity. SARS-CoV-2 internalization occurs via S-protein-mediated cell binding followed either by direct fusion with the plasma membrane or endocytosis and subsequent fusion with the endosomal membrane. Despite the crucial role of virus internalization, the precise kinetics of the processes involved remains elusive. We developed a pipeline, which combines live-cell microscopy and advanced image analysis, for measuring the rates of multiple internalization-associated molecular events of single SARS-CoV-2-virus-like particles (VLPs), including endosome ingression and pH change. Our live-cell imaging experiments demonstrate that only a few minutes after binding to the plasma membrane, VLPs ingress into RAP5-negative endosomes via dynamin-dependent scission. Less than two minutes later, VLP speed increases in parallel with a pH drop below 5, yet these two events are not interrelated. By co-imaging fluorescently labeled nucleocapsid proteins, we show that nucleocapsid release occurs with similar kinetics to VLP acidification. Neither Omicron mutations nor abrogation of the S protein polybasic cleavage site affected the rate of VLP internalization, indicating that they do not confer any significant advantages or disadvantages during this process. Finally, we observe that VLP internalization occurs two to three times faster in VeroE6 than in A549 cells, which may contribute to the greater susceptibility of the former cell line to SARS-CoV-2 infection. Taken together, our precise measurements of the kinetics of VLP internalization-associated processes shed light on their contribution to the effectiveness of SARS-CoV-2 propagation in cells.

## 1. Introduction

COVID-19, caused by the SARS-CoV-2 virus, led to a major disruption of everyday life, demonstrating how zoonotic events can occur unexpectedly and have a catastrophic impact on the general population [1,2]. At present, more than 774 million cases have been reported, of which more than 7 million have been reported to be fatal (WHO). Part of the β-coronavirus family [3,4], SARS-CoV-2 is a positive-sense single-stranded RNA virus harboring 14 ORFs that encode 27 proteins [5]. These include structural proteins, namely, the S (spike), M (membrane), N (nucleocapsid), and E (envelope), which play major roles in viral survival, propagation, infectivity, and virulence [6,7]. Different systems have been developed to recreate the SARS-CoV-2 infection process in low-biosafety lab conditions. A prominent approach is the use of virus-like particles (VLPs) [8,9,10,11,12,13] which self-assemble in cells after expression of the SARS-CoV-2 structural proteins. VLPs faithfully recapitulate the step of viral entry, while unable to replicate, which renders them non-infectious [8]. SARS-CoV-2 VLP formation is driven primarily by the M protein, while the E protein plays a potentiating role [10,12]. While these two proteins are sufficient to form a particle, the S protein is required for entry into host cells. Interestingly, the addition of S to SARS-CoV-2 VLPs leads to a decrease in the amount of M and E. The optimal proportion of structural proteins M, N, E, and S for the production of stable SARS-CoV-2 VLPs is 3:12:2:5 [10,12]. Further, addition of a specific cis-acting RNA element derived from SARS-CoV-2 to the VLPs increased packaging efficiency [8].

Two mechanisms, namely, membrane fusion and endocytosis, have been demonstrated to play a role in SARS-CoV-2 entry into cells [14]. SARS-CoV-2 binds to target cell membranes through the interaction of S with cell membrane proteins (SR-B1, AXL, KIM1/TIM1, CD147, Neuropilin-1,2, DC-SIGN, L-SIGN, and others), the most prominent of which is angiotensin-converting enzyme 2 (ACE2) [14,15,16,17,18,19,20,21,22,23,24,25]. The considerable number of proteins participating in the recognition of SARS-CoV-2 account for its wide tissue and cell tropism. The ACE2-bound S protein is recognized by the membrane-bound transmembrane serine protease 2 (TRMPRSS2), which cleaves the S protein at the S2′ site, leading to a dramatic conformational change that allows fusion between the virus and host cell membranes [14]. However, it was reported that fusion takes place in the absence of ACE2 when tethering between the VLPs, and liposome membranes occurs, with the presence of ACE2 only stimulating the process [26] Membrane fusion results in the release of viral genetic information into the cell, setting the stage for viral replication [27]. If the S protein–receptor complex is not engaged by this protease due to a low membrane concentration or absence of the latter, ACE2-bound SARS-CoV-2 is internalized via clathrin-mediated endocytosis [28]. The pH of the virus-containing endosomes then drops, leading to activation of the cathepsin L protease [29]. Cathepsin L-mediated cleavage of SARS-CoV-2 at the S2′ site enables fusion of the viral and endosome membranes as well as nucleocapsid release into the host cell [30].

Recent real-time imaging studies provided valuable insights into the process of SARS-CoV-2 internalization. Tracking of single vesicular stomatitis virus (VSV) chimeras containing the SARS-CoV-2 S protein revealed that SARS-CoV-2 entry requires an acidic environment [31,32]. A detailed understanding of viral entry, however, requires a comparison of kinetic parameters of VLP internalization to those of acidification and endosomal ingress, which requires VLPs possessing all four SARS-CoV-2 structural proteins.

Herein, we employed live-cell imaging and a dedicated image analysis pipeline (Single-Particle Tracking Analysis in Cells Using Software Solutions, SPARTACUSS 1.0) to precisely follow and quantify the timing of VLP internalization, VLP-containing endosome ingression, acidification, active microtubular transport, and nucleocapsid release. Our temporal characterization reveals the sequence and interdependence of the above-described processes. Rapid VLP acidification, which coincides with dynamin-mediated endosome scission and nucleocapsid release, occurs 4 and 12 min after plasma membrane binding in VeroE6 and A549 cells, respectively, quickly followed by the initiation of active microtubule-dependent VLP motion. Our results suggest that VLP fusion (nucleocapsid release) occurs in parallel to or shortly after endosome formation. Surprisingly, the VLPs do not co-localize with early endosomes during VLP internalization. The more rapid internalization observed in VeroE6 cells may contribute to the infectivity of SARS-CoV-2 observed in these cells relative to A549. Further, neither Omicron or del-1 mutations influence the kinetics of SARS-CoV-2 VLP internalization.

## 2. Materials and Methods

### 2.1. Cell Lines and Transfections

The VeroE6, U2OS, and A549 cells used in this study were obtained from American Type Cell Culture (ATCC), Manassas, VA, USA. VeroE6-ACE2-TMPRSS2 (VeroE6-AT) and A549-ACE2 clone 8-TMPRSS2 (A549-AT) cells, which overexpress ACE2 and TMPRSS2, were provided by the NIBSC Research Reagent Repository, Hertfordshire, UK (catalogue numbers 101003 and 101006, respectively) and were a kind gift from Prof. Arvind Patel, University of Glasgow. VeroE6 and U2OS cell lines were cultured in Dulbecco’s Modified Eagle Medium (DMEM, Thermo Fisher Scientific, Waltham, MA, USA) with a high content of glucose, 10% fetal bovine serum (FBS), and 100 units/mL penicillin and 100 μg/mL streptomycin at 37 °C and 5% CO_2_. A549 cell lines were cultured in Roswell Park Memorial Institute medium (RPMI, Thermo Fisher Scientific, Waltham, MA, USA) supplemented with 10% FBS (Thermo Fisher Scientific, Waltham, MA, USA) 100 units/mL penicillin (Thermo Fisher Scientific, Waltham, MA, USA) and 100 μg/mL streptomycin (Thermo Fisher Scientific, Waltham, MA, USA) at 37 °C and 5% CO_2_. In addition, 2 mg/mL Geneticin (Thermo Fisher Scientific, Waltham, MA, USA) and 200 µg/mL Hygromycin B (InvivoGen, San Diego, CA, USA) were added to the culture medium of VeroE6-AT and A549-AT cells, respectively.

### 2.2. Transfection

For expression of Ace2NeonGreen and mNeonGreen, we performed transient transfection via baculovirus-mediated gene transduction of mammalian cells (BacMam) using the Montana Molecular ACE2 green kit (C110G, Bozeman, MT, USA) as per the manufacturer’s protocol. Expression of the fluorescent proteins was evaluated via fluorescence microscopy two days after transfection, whereafter cells were treated with VLPs.

For expression of eGFP-tagged dynamin 1, we used a plasmid that was a gift from Sandra Schmid (Addgene plasmid # 34680; http://n2t.net/addgene:34680; RRID: Addgene_34680) [27]. To inhibit the dynamin-mediated entry into cells we used Dynole^®^ 34-2, a dynamin I/II inhibitor (ab120463, Abcam, Cambridge, UK). Cells were treated with 30 µmol Dynole for 15 min and were then imaged every 30 s in 31 Z-planes, with a Z-step size of 0.2 µm.

To observe endosome trafficking, we used CellLight™ Early Endosomes-GFP, BacMam 2.0 (Catalog number: C10586, Thermo Fisher Scientific, Waltham, MA, USA), which allowed us to introduce GFP-tagged Rab5a into cells and thus visualize early endosome vesicles. We plated 10,000 cells in 35 mm glass-bottom culture dishes (MatTek Corporation, Ashland, MA, USA) and incubated these with 2 µL of CellLight™ Early Endosomes-GFP overnight. On the following day, we added VLPs and imaged the cells at 30 s intervals in 11 Z-planes with a Z-step size of 0.2 µm.

To observe late endosomes, we used LysoTracker (ThermoFisher Scientific, Waltham, MA, USA). Cells were incubated with the VLPs for 30 min, treated with 50 mM Lysotracker for 1 min, and imaged every 30 s in 11 Z-planes with a Z-step size of 0.2 µm.

To image tubulin, we used abberior LIVE 610 conjugated to cabazitaxel. Cells were inoculated with VLPs for 30 min and incubated with abberior LIVE 610 conjugated to cabazitaxel for 15 min, followed by washing with fresh FluoroBrite™ DMEM (ThermoFisher Scientific, Waltham, MA, USA). The cells were imaged every 30 s in a single Z-plane.

### 2.3. Time-Lapse Live-Cell Imaging

Forty-eight hours before imaging, all cells were transferred to MatTek glass-bottom dishes (MatTek Corporation, Ashland, MA, USA) at 20% confluence. Live-cell imaging was performed on an Andor Dragonfly spinning-disk confocal system with a Nikon Eclipse Ti2-E inverted microscope equipped with the Nikon Perfect Focus System (PFS), a Nikon CFI Plan Apo VC 60× (NA 1.2) water immersion objective, a Nikon Apo 60× (NA 1.4) oil objective, or a Nikon HP Plan Apo 100× (NA 1.35) silicone λS objective, and a high-sensitivity iXon 888 Ultra Electron Multiplying Charge-Coupled Device (EMCCD) camera. Time intervals between consecutive frames varied between 15 and 30 s depending on the type of experiment, cell line, and the labeled protein. Images were acquired with variable z-stacks of between 1 and 36 steps depending on the type of experiment and a z-step size of 0.2 µm. Prior to imaging, Petri dishes mounted on the microscope were left to thermally equilibrate for at least 30 min. All cells were incubated in FluoroBrite™ DMEM (ThermoFisher Scientific, Waltham, MA, USA) for imaging and maintained at 37 °C and 5% CO_2_ during imaging. To visualize cells as transparent and opaque, we used the Imaris 9.6.1 imaging software tool (Oxford Instruments, Abingdon, UK).

### 2.4. Electron Microscopy

MLE-12 cells (a gift from Dr Kristi Warren) were grown on ACLAR disks and incubated with SARS-CoV-2 VLP^Wu^ for 5 min. Cells were fixed in 2.5% glutaraldehyde plus 1% paraformaldehyde in 0.1 M cacodylic buffer for 30 min and then embedded in resin using an Embed 812 kit (Electron Microscopy Sciences, Hatfield, PA, USA) and sectioned at 80 nm with a diamond knife (Diatome, Nidau, Switzerland) using a Leica EM UC6 (Leica Microsystems, Wetzlar, Germany). Sections were visualized using a JEM 1400 Plus electron microscope (JEOL, Tokyo, Japan) at 120 kV.

### 2.5. Virus-like Particle Preparation

VLPs were prepared as previously described [11,12]. Once generated, these were kept on ice until use 1 to 6 days after production.

### 2.6. Antibody Inhibition of SASR-CoV-2 VLPs

We diluted VLPs in 25 µL of FluoroBrite™ DMEM (ThermoFisher Scientific, Waltham, MA, USA) without FBS and then added 2 µL of Recombinant Anti-SARS-CoV-2 Spike Glycoprotein S1 antibody [CR3022] (ab273073, Abcam, Cambridge, UK). After incubating this mixture for 30 min, we added it to cells and proceeded with image acquisition.

### 2.7. VLP Tracking

The channel containing VLP fluorescence was isolated in Fiji and smoothed with 3D Gaussian filtering (1.5-pixel radius in XY and Z). Maximum intensity projection (MIP) was then performed.

The particles were tracked using the MTrackJ plugin, clicking on the particle location in each time frame, using the option “Apply local cursor snapping during tracking” with a range of 5 × 5 pixels. Completed tracks were exported as .mdf files. Multiple particles were tracked in the same session. To continue previous tracking sessions, we imported the previous .mdf file and continued, so that one .mdf file contained all the tracks of one movie.

The subsequent processing steps outlined below were performed with a set of Python scripts and Fiji macros.

To convert the 2D + t tracks to 3D + t, the script adds the Z coordinate by going back to the 3D movie before MIP and finding the plane that contains the maximum signal along a cylinder with a 2-pixel radius, centered at the location of the particle.

Instantaneous VLP speed v(t) at each time t was measured by subtracting the positions of a particle at times (t + 1) and t, then dividing by the time interval between successive frames. The positions were converted to real physical units (nanometers) using the pixel sizes in XY and Z.

To visualize single particles over time, a cuboid with size dx, dy, and dz was cropped out of the 3D image stack for each time point, such that the particle was located at the center of the square (dx, dy), while dz was equal to the full width of the stack. Thus, the motion of the particle in Z could be visualized. The cuboids were maximum-projected along either the X or Y axis, resulting in ZY or ZX rectangles, respectively. For completeness, we also generated the XY squares, by performing max-Z projections. The stacking of these three ZY, ZX, and XY crops is referred to as a kymograph and discussed throughout the paper.

### 2.8. VLP Analysis

After having information on the position, speed, and intensity of each particle, we determined the precise moment when the intensity started to decrease and the speed started to increase—specific hallmarks of the VLP internalization process. Every particle was then aligned to these positions, and an average for the speed and intensity was obtained. For every intensity value, a measured background value was subtracted. The curves were then plotted and compared showing the standard deviation for each point as error bars.

When determining the pH of each particle, we used the intensity values and transformed them to pH values using the following formula: 7.11 − log10(1/(X × 0.886) − 1) [33], where X stands for the corresponding intensity value.

### 2.9. Statistical Analysis

Between-group comparisons were performed using Student’s *t*-test. The significance threshold was set at *p* < 0.01. Data are presented as the mean ± standard deviation.

## 3. Results

### 3.1. Visualization of VLP Internalization

**Figure 1:**
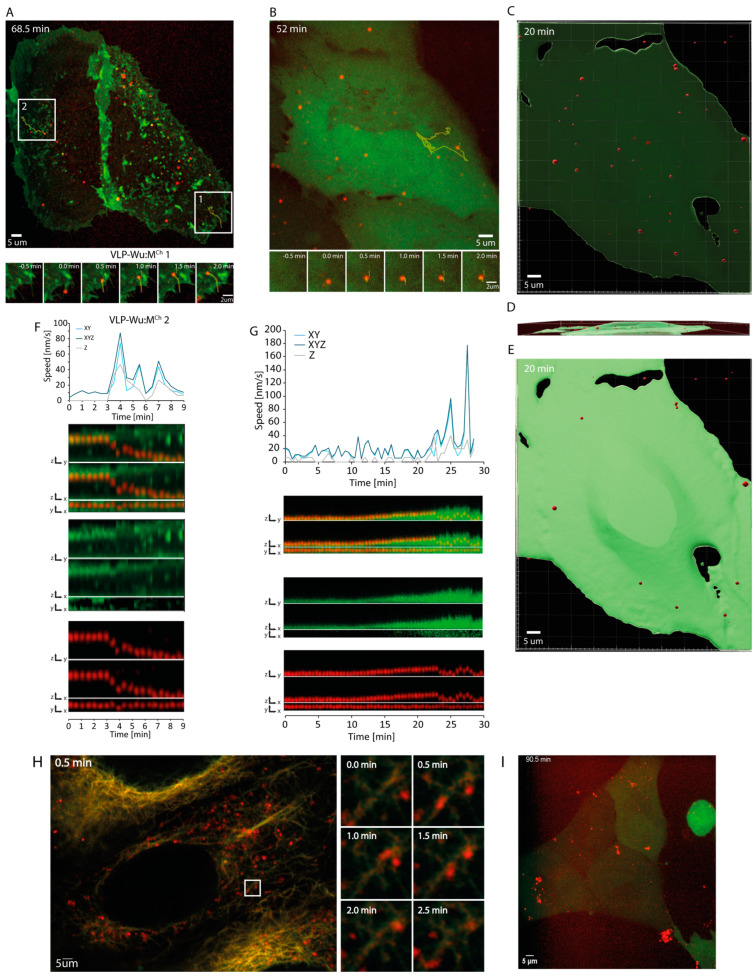
Visualization of SARS-CoV-2 VLPs’ binding and internalization into host cells. (**A**) Binding and internalization of SARS-CoV-2 VLP^Wu^:M^Ch^ into U2OS cells overexpressing ACE2-Neon green and TMPRSS2. The montage shows VLPs binding to filopodia first and then migrating into the cell body. (**B**) U2OS cells expressing mNeonGreen (to visualize cell volume) treated with VLP^Wu^:M^Ch^. (**C**) The positions of VLP^Wu^:M^Ch^ in the transparent 3D volume of a U2OS cell (viewed from the top). (**D**) The cell from (**C**) shown from the side. (**E**) Same as (**C**) except VLP^Wu^:M^Ch^ are shown in a volumized U2OS cell to highlight internalized VLPs (viewed from the top). (**F**) Graph showing the speed of the VLP (2) from (**A**); each point in the graph represents the speed of the particle over time extracted from two consecutive images in 1D—vertically (Z), 2D (XY), and 3D (XYZ). The kymograph represents the change in intensity of the particle and its position in Z as described in detail in Figure S1. The time scales of the graph and kymographs are aligned. (**G**) Same as (**F**), but for the particle in (**B**). (**H**) Cells treated with Tubulin 610 conjugated to cabazitaxel, showing the transport of VLP^Wu^:M^Ch^ via microtubules. (**I**) VLP^Wu^:M^Ch^ treated with a Recombinant Anti-SARS-CoV-2 S1 antibody. The VLPs aggregate and are unable to internalize into cells.

To study the kinetics of SARS-CoV-2 entry into cells, we employed VLPs derived from HEK293 cells overexpressing SARS-CoV-2 structural proteins M, E, and S of the Wuhan variant [11]. These VLPs successfully recapitulate SARS-CoV-2 internalization [10,12], binding to the host cell surface via S protein, whereafter they are internalized, as shown by a TEM micrograph of thin-sliced MLE-12 cells in the process of endocytosing a SARS-CoV-2 VLP^Wu^ (Figure S1). To visualize internalization, we used VLPs containing M-mCherry (as well as unlabeled M) [12]. Hereafter, VLP^Wu^:M^Ch^ is short for VLP^Wuhan^:(E, S, M&M-mCherry). We treated U2OS cells which overexpress NeonGreen-tagged ACE2, non-labeled ACE2, and TRMPRSS2 with these VLPs and visualized the movement of particles in 3D at 30 s intervals via multi-point spinning-disk live-cell microscopy. We observed adherence of the VLPs to the cell membrane, often on the filopodia, as previously suggested [34] (Figure 1A, Videos S1–S3). We manually tracked each particle after membrane binding in order to acquire its speed and position in 3D (see Section 2). Interpreting these multidimensional data requires clear and intuitive visualization, as well as precise measurement. To this end, we coupled the tracking procedure to a post-processing pipeline for extracting and visualizing the multidimensional tracking results for each single VLP. We named this procedure SPARTACUSS (Single-PARTicle Tracking Analysis in Cells Using Software Solutions).

For the visualization of a single particle via SPARTACUSS, we first cropped the immediate space around the particle’s x and y coordinates while keeping the entire z-stack; additionally we used Gaussian blur to make the VLPs more distinct (Figure S2A). Next, we performed maximum intensity projections as follows: maximum intensity of x—to show the particle’s position in z and y; maximum intensity of y—to visualize the particle’s position in z and x; and maximum intensity of z—to depict the particle’s position in x and y. We repeated this for each time point and combine the crops in a one-block kymograph (Figure S2B,C). Finally, we created such a block for the 488 channel (Green) and 591 mCherry (Red) channel, and a merged block for both channels (Figure S2C). SPARTACUSS allows us to measure and plot the speed in 3D (x,y,z), as well as the changes in the intensity of the labeled structural proteins in individual VLPs (Figure S2C).

Using the SPARTACUSS workflow, we observed that, after binding, the VLPs initially moved slower (10–20 nm/s), whereafter their speed increased. This speed increase frequently coincided with downward motion in z, suggestive of particle internalization (Figure 1A,B,F,G, Videos S1–S7).

To confirm if this downward motion indeed marks VLP entry into cells, we expressed mNeonGreen, which freely diffuses throughout the whole cell volume, using it to reconstruct the cells in 3D (Videos S4–S6). To see both the membrane-bound and internalized VLPs, we use transparent visualization of the mNeonGreen cell volume (Figure 1C, Video S7). In order to distinguish between the two VLP populations, we make the volume opaque (Figure 1E, Video S7), which renders most VLPs not visible once inside the cell. Internalized VLPs can be subsequently visualized using a side view of the transparent cell volume image (Figure 1D, Video S7). Visualization of a single VLP confirms that its speed increase in 3D coincides with cell surface penetration, as observed with the ACE2 tagging discussed above (Figure 1B,G). These results clearly demonstrate the capability of SPARTACUSS to capture the exact moment of VLP entry into cells and thus measure its 3D dynamics in real time.

To understand if active transport along microtubules underpins the increased movement of particles once inside the cell, we pre-incubated cells with VLP^Wu^:M^Ch^, treated them with abberior LIVE 610 tubulin dye (cabazitaxel conjugated with LIVE 610 dye) for 10 min, and immediately proceeded with live-cell imaging. We observed continuous co-localization between VLPs and microtubules (Figure 1H), indicating that fast particle movement occurs along the microtubule network (Video S8).

Next, we sought to assess how anti-SARS-CoV-2 S protein antibodies neutralize the SARS-CoV-2 virus. To this end, we incubated cells with VLP^Wu^:M^Ch^ pre-treated with an Anti-SARS-CoV-2 Spike S1 (CR3022 clone) antibody. Antibody pre-incubation precipitated most of the VLPs, preventing cell entry by effectively reducing their free concentration (Figure 1I and Video S9).

### 3.2. Dynamin-Dependent VLP Entry

**Figure 2:**
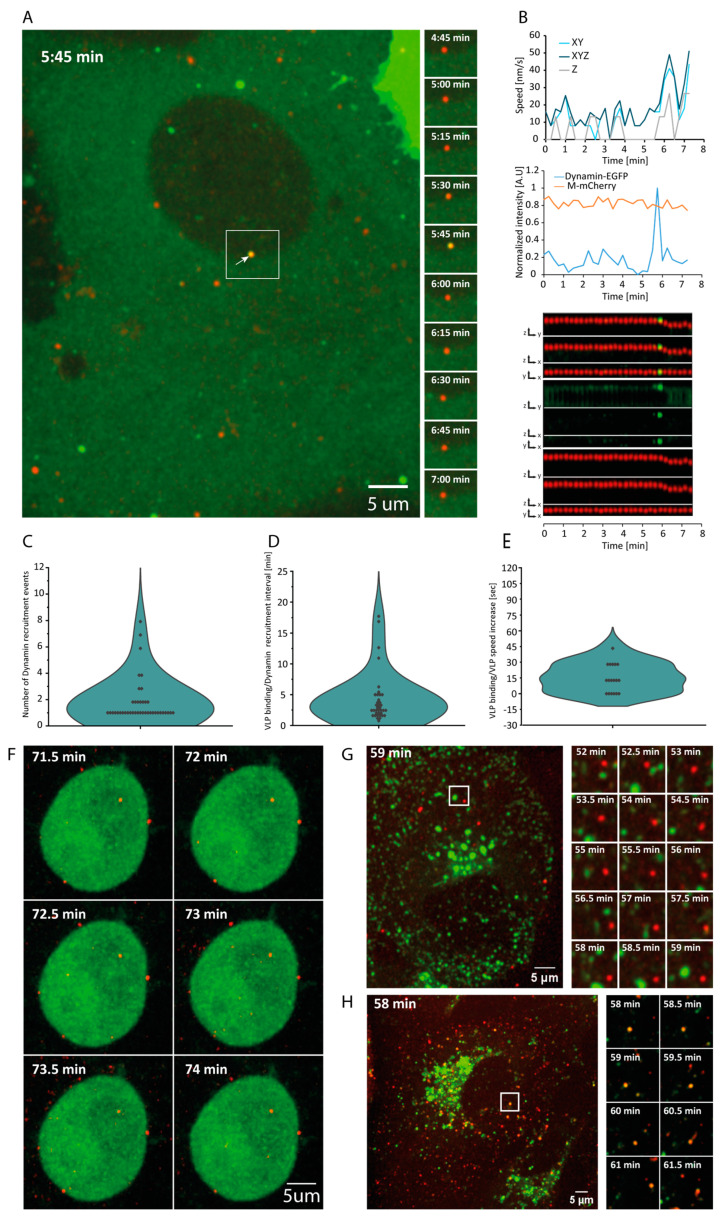
Dynamics of dynamin recruitment to the site of SARS-CoV-2 VLP^Wu^:M^Ch^ binding in Vero E6 cells. (**A**) A representative image of SARS-CoV-2 VLP^Wu^:M^Ch^ added to Vero E6 cells and dynamin recruitment. The montage shows consecutive images from the area shown by the white square; recruitment of dynamin is observed at 5:45 min (white arrow). (**B**) The top graph represents the speed of the VLP shown in (**A**); the bottom graph demonstrates the dynamics of the intensity of both channels; the kymograph represents the change in intensity and position of the particle. (**C**) Distribution of the number of dynamin recruitment events to bound VLP^Wu^:M^Ch^, n = 41. (**D**) Distribution of time intervals between VLP^Wu^:M^Ch^ binding and the first recruitment of dynamin, n = 37. (**E**) Distribution of the time intervals between VLP^Wu^:M^Ch^ binding and VLP speed increase, n = 18. (**F**) Cells treated with Dynole 34-2, which inhibits vesicle-mediated endocytosis, showing the inability of VLPs to enter cells. (**G**) Cells expressing GFP-tagged Rab-5 showing lack of co-localization of the Rab5-positive vesicles with the VLP^Wu^:M^Ch^. (**H**) Cells treated with LysoTracker showing co-localization of the VLP^Wu^:M^Ch^ with acidic vesicles (lysosomes or late endosomes).

Endocytosis has been suggested to play a role in VLP entry [35,36]. To determine the duration of time between VLP binding and ingress, we expressed GFP-tagged dynamin in Vero E6 cells which are highly susceptible to SARS-CoV-2 infection [37]. Dynamin binds to the invaginated clathrin-coated vesicles for 10 to 15 s and is responsible for vesicle scission during endocytosis [38,39,40]. The transient dynamin foci formed during this process are a standard marker of endosome formation [38,41,42,43]. Time-lapse imaging of dynamin-1-GFP-expressing cells treated with VLP^Wu^:M^Ch^ allowed us to visualize VLP movement across the cells as well as the “blinking” of short-lived dynamin foci, indicative for endosome ingress (Videos S10–S12). A share of 49% of the VLPs co-localized with dynamin foci during or immediately after the above-described speed increase and downward movement (Figure 2A,B). This transient co-localization strongly suggests that endocytosis is involved in VLP internalization.

Dynamin accumulation does not always reflect successful scission of the endocytic vesicles, and several abortive cycles are often observed before a productive scission occurs [44]. If a single VLP co-localizes transiently with dynamin more than once, it would be indicative of such abortive events. Indeed, we observed that 64% of the particles co-localized with a dynamin focus only once, 17% co-localized twice, and 19% experienced three or more co-localization events. These results suggest that in 36% of VLP entries, there is at least one abortive dynamin binding (Figure 2C). In addition, dynamin foci formation allowed us to measure the time between VLP binding to the membrane and endosome vesicle scission, which was 5.24 ± 6.8 min. SPARTACUSS also allowed us to measure the time between dynamin foci formation and VLP speed increase. Particles increased their speed within 45 s of dynamin binding, and, in 30% of cases, this increase occurred in parallel with the dynamin scission event (Figure 2D,E).

To further evaluate the role of endocytosis in VLP internalization, we used Dynol 34-2, a potent inhibitor of dynamin 1, which prevents receptor-mediated endocytosis [43]. After Dynol 34-2 treatment, VLPs did bind to the cell surface but did not enter (Figure 2F, Video S13). However, it should be noted that Dynol 34-2 also greatly altered cell morphology, inducing a round phenotype, indicative of an effect on the cell cortex. Such considerable changes in morphology could affect membrane-related processes, including endocytosis. To check if VLPs localized within the early endosome, we used cells expressing Rab5a-GFP. Surprisingly, we did not detect co-localization of the VLPs with early endosomes (Figure 2G and Video S14). As endosome maturation is paralleled by a drop in pH, we asked whether the VLPs co-localize with acidic vesicles [45]. To this end, we stained cells with LysoTracker, which marks acidic vesicles, after incubation of cells with VLPs. Some of the VLPs co-localized with the stained acidic vesicles (Figure 2H and Video S15).

Taken together, our results indicate that dynamin-mediated endocytosis is involved in VLP internalization. Furthermore, VLPs are not internalized via Rab5a-positive early endosomes but rather localize within acidic vesicles.

### 3.3. Dynamics of VLP Acidification

**Figure 3:**
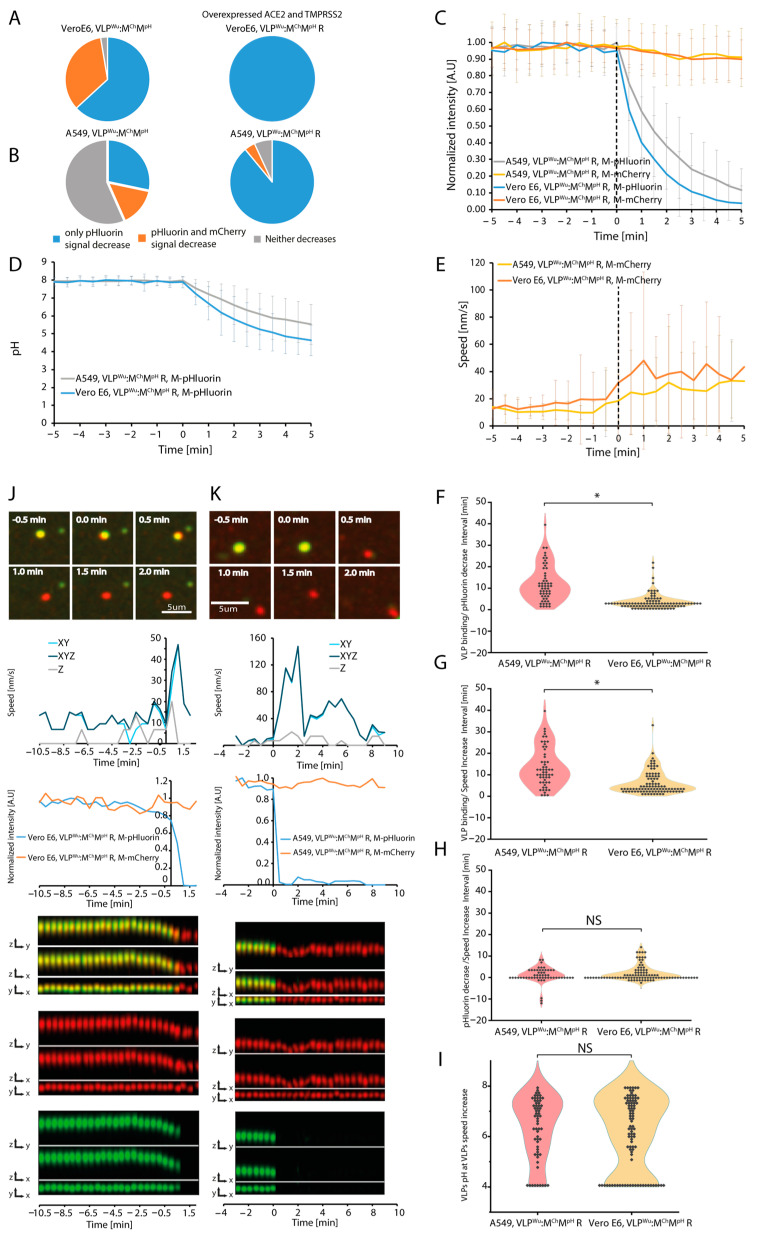
Dynamics of SARS-CoV-2 VLP^Wu^:M^Ch^M^pH^R binding, pH decrease, and speed increase in A549 and Vero E6 cells. (**A**) Percentage of VLPs in which only M-pHluorin intensity decreases (blue), the intensities of M-pHluorin and M-mCherry decrease simultaneously (orange), or neither decreases (gray) for 100 min after addition of VLP^Wu^:M^Ch^M^pH^R in VeroE6 cells with or without ACE2 and TMPRSS2 overexpression. (**B**) Same experiment as (**A**) in A549 cells. (**C**) Comparison of the M-pHluorin intensity decrease during internalization of VLP^Wu^:M^Ch^M^pH^R in A549 and Vero E6 cells. The average intensity of pHluorin is represented as a function of time where the individual VLPs were aligned to the start of VLP pHluorin decrease (0 min). The average M-mCherry intensity of the same particles is also presented. Errors bars represent the standard deviation. For A549 n = 55, for Vero E6 n = 93. (**D**) Comparison of the dynamics of pH decrease during internalization of VLP^Wu^:M^Ch^M^pH^R in A549 and Vero E6 cells. The pH decrease is calculated based on the measured M-pHluorin intensity decrease. The average pH of VLPs is represented as a function of time where individual VLPs are aligned to the start of VLP pHluorin decrease (0 min). Error bars represent the standard deviation. For A549 n = 55, for VeroE6 n = 93. (**E**) The average speed of VLP^Wu^:M^Ch^M^pH^R in A549 and Vero E6 cells measured based on the tracked M-mCherry signal. The average speed of VLPs was calculated after alignment of the individual VLP speeds to the start of the VLP pHluorin signal decrease (0 min). Error bars represent the standard deviation. For A549 n = 55, for Vero E6 n = 93. (**F**) Distribution of time intervals between VLP^Wu^:M^Ch^M^pH^R binding and the start of pHluorin intensity decrease in A549 cells or Vero E6 cells. Two-tailed Student’s *t*-test; * *p* < 0.01. For A549 n = 55, for Vero E6 n = 93. (**G**) Distribution of time intervals between VLP^Wu^:M^Ch^M^pH^R binding and the start of VLP speed increase in A549 and Vero E6 cells. Two-tailed Student’s *t*-test; * *p* < 0.01. For A549 n = 55, for Vero E6 n = 93. (**H**) Distribution of time intervals between the start of VLP^Wu^:M^Ch^M^pH^R pHluorin intensity decrease and the start of VLP speed increase in A549 and Vero E6 cells. Two-tailed Student’s *t*-test; NS *p* > 0.01. For A549 n = 55, for Vero E6 n = 93. (**I**) Distribution of the estimated pH of VLP^Wu^:M^Ch^M^pH^R, calculated based on the pHluorin signal at the moment when VLP speed started to increase in A549 and Vero E6 cells. Two-tailed Student’s *t*-test; NS *p* > 0.01. For A549 n = 55, for Vero E6 n = 93. (**J**) Representative time-lapse images (top), corresponding VLP speed and intensity graphs (middle), and kymographs (merged, M-mCherry, and M-pHluorin) in all dimensions (bottom) for a single VLP^Wu^:M^Ch^M^pH^R undergoing internalization in an VeroE6 cell. In this example, the speed increases in parallel with pHluorin signal decrease. (**K**) Same as (**J**) in a A549 cell.

The pH of medium was previously shown to influence the internalization of an SARS-CoV-2 S protein-containing VSV chimera, with a more acidic environment promoting internalization preferentially via fusion [46,47,48]. To evaluate the role of pH in VLP internalization, we sought to precisely measure the pH dynamics of VLPs in which a fraction of the M protein is tagged with superecliptic pHluorin in its C-terminal end [33]. This protein emits bright fluorescent light at pH ≥ 8, but its intensity sharply decreases at pH < 7.5, completely disappearing at pH < 5 [49]. As the M protein C-terminal domain lies inside the VLPs, the fused pHluorin serves as a real-time indicator of the intra-VLP pH. The pHluorin-labeled VLPs in the medium (pH8) emitted a bright green signal; however, shortly after binding to the membrane of Vero E6 cells, the intensity rapidly disappeared (Video S16), suggesting that the pH of VLPs decreases sharply. To understand when this happens relative to VLP internalization, we used VLPs, which contain unlabeled E, S, N, and M proteins but also two labeled fractions of M protein—one with mCherry and another with pHluorin, in addition to the cis-acting RNA element [8]. We will refer to these VLPs as VLP^Wu^:M^Ch^M^pH^ (short for VLP^Wuhan^:(E, S, N, M&M-mCherry&M-pHluorin). The dual VLP labeling enabled us to follow the VLP and analyze its dynamics even when complete disappearance of the pHluorin signal occurred due to a sharp drop in pH. We observed that in 63% of the VLPs, the pHluorin signal disappeared without any change in mCherry intensity, while in 34% of the VLPs both fluorescent signals disappeared simultaneously, and in 3% of the particles there was no change in the fluorescent signal during the time course of our experiment in Vero E6 cells (Figure 3A). These results indicate that in two-thirds of the cases, the disappearance of the pHluorin fluorescent signal is a result of pH decrease without VLP disassembly.

Next, we studied the influence of concurrent ACE2 and TMPRSS2 protease overexpression on VLP internalization in Vero E6 cells. To this end, we used VLPs containing RNA (VLP^Wu^:M^Ch^M^pH^, R), which included the cis-acting T20 element reported to enhance packaging [8]. The inclusion of this RNA element did not affect the percentages of VLPs, in which the pHluorin signal disappeared without any change in mCherry intensity (Figure 3. Meanwhile, ACE2 and TMPRSS2 overexpression in cells led to a significant increase in the number of such VLPs (up to 100%) (Figure 3A). Next, we performed the same experiments with human lung adenocarcinoma A549 cells, which are the standard pulmonary epithelial cell model for SARS-CoV-2 infection (Video S17). Without ACE2 and TMPRSS2 overexpression, 28% of the VLPs exhibited a decrease in pHluorin fluorescence without any change in mCherry intensity, 15% exhibited simultaneous disappearance of both signals, and 57% did not show any change in both signals throughout the experiment (Figure 3B). These results suggest that A549 cells are less susceptible to SARS-CoV-2 VLP internalization than Vero E6 cells. Overexpression of ACE2 and TMPRSS2 considerably increased the fraction of VLPs exhibiting a decrease in pHluorin intensity, without changes in mCherry intensity (89% versus 28%) (Figure 3B). Overall, ACE2 and TMPRSS2 overexpression increased the number of VLPs exhibiting a decrease in pH without VLP disassembly in both cell lines (Figure 3A,B).

To evaluate the speed with which the pHluorin signal decreases, we aligned all VLP tracks to the start of the pHluorin intensity decrease (Figure 3C). We thus measured the half-time of pHluorin signal disappearance, which was 1.4 and 1.6 min in Vero E6 and A549 cells, respectively. Conversion of pHluorin intensity to pH values [33] showed that the VLP pH in Vero E6 cells decreased from 8 to 6.3, while that in A549 cells decreased from 8 to 6.9 over a period of 1.5 min (Figure 3C,D), attesting to the rapid acidification of VLPs. Our results also demonstrated that the speed of VLPs tends to increase following the start of pH decrease (Figure 3E).

Dual labeling with both M-mCherry and M-pHluorin allowed us to infer the temporal order of the three stages of VLP internalization at the single VLP level: VLP binding to the cell membrane, VLP pH decrease, and the increase in its speed. We thus measured the time intervals between each possible pair of the above-described internalization steps (Figure 3F–H, and Table 1). In VeroE6 cells, VLP pH began to decrease 4.1 ± 3.6 min after plasma membrane binding, and 2.4 ± 3.7 min later the VLP speed started to increase (Table 1). In A549 cells, VLP pH began to decrease 12.5 ± 8.4 min after binding, and 1.4 ± 3.7 min later the VLP speed started to increase. These results indicate that, on average, the pH change occurs during or immediately before the VLP speed increases (Figure 3J,K). At a single VLP level, however, we have examples where the speed increase occurs after the pH decreases, but also examples where the speed increases simultaneously or before the start of pH decrease (Figures S3 and S4, and Videos S18–S21), indicating that there is no direct causal relationship between the two events. This uncoupling is further supported by the significant variation in pH at which the VLP speed increase begins (Figure 3I). As we demonstrate above, the speed increase is a hallmark for active microtubule movement of the vesicles containing VLPs. Taken together, for the majority of VLPs, acidification starts before or during microtubule attachment (Figure S3C). As acidification takes less than 2 min, we observe cases when movement via the microtubules occurs after the pH is already <5. Direct comparison of VLP internalization kinetics among the two cell lines (Figure 3H) revealed no statistically significant difference in the interval between VLP pH decrease and speed increase (start of the microtubule movement). However, the intervals between VLP binding and both pH decrease and speed increase are 2–3 times shorter in VeroE6 cells than in A549 cells (Figure 3, Table 1). This difference in internalization efficiency may contribute to the greater SARS-CoV-2 susceptibility of Vero E6 relative to A549 cells, generally attributed to the lack of interferon signaling in the former [50,51,52,53]. Close examination of the distribution of the intervals between VLP binding and both pH decrease and speed increase in A549 cells revealed a small population of VLPs for which these intervals are significantly longer (Figure 3F,G). These longer intervals may be attributed to cycles of abortive dynamin-mediated endosome scission, as previously discussed. Importantly, ACE2 and TMPRSS2 overexpression had no effect on the dynamics of VLP speed increase and pH decrease in both Vero E6 and A549 cells (Figures S5 and S6), despite enhancing VLP uptake efficiency (Figure 3A,B).

In summary, our findings highlight the notable speed and efficacy of VLP internalization, which are more pronounced in VeroE6 relative to A549 cells. Furthermore, the dynamic profiles of VLP internalization-related processes, namely, membrane binding, acidification, and initiation of microtubule transport, show that the latter two processes, while temporally proximal, are not interdependent.

### 3.4. Kinetics of VLPs Lacking the Furin Cleavage Site

**Figure 4:**
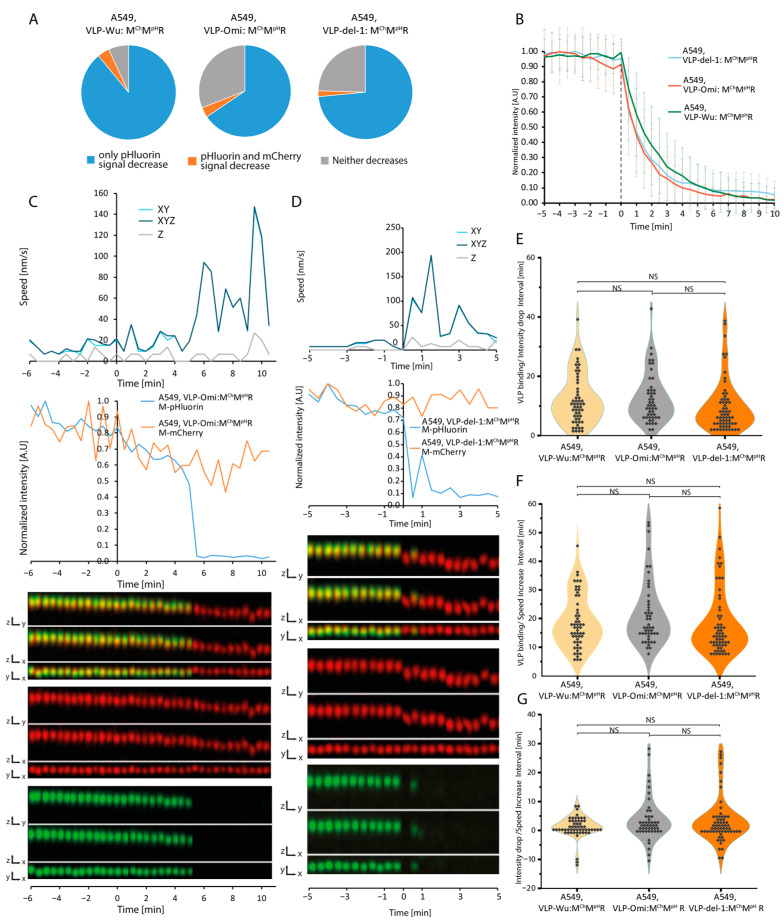
Comparison of VLP binding, acidification, and speed increase dynamics between VLP^Wu^:M^Ch^M^pH^R, VLP^Omi^:M^Ch^M^pH^R, and VLP^del-1^:M^Ch^M^pH^R during internalization in A549 cells. (**A**) Percentage of VLPs in which only the M-pHluorin intensity decreases (blue), M-pHluorin and M-mCherry intensities decrease simultaneously (orange), or neither decreases (gray). (**B**) Comparison of pHluorin intensity decrease during the internalization of VLP^Wu^:M^Ch^M^pH^R, VLP^Omi^:M^Ch^M^pH^R and VLP^del-1^:M^Ch^M^pH^R in A549 cells. The average intensity of pHluorin is represented as a function of time where the individual VLPs were aligned to the start of VLP pHluorin decrease (0 min). The average M-mCherry intensity is also presented. Error bars represent the standard deviation. For VLP^Wu^:M^Ch^M^pH^R n = 55, for VLP^Omi^:M^Ch^M^pH^R n = 48, for VLP^del-1^:M^Ch^M^pH^R n = 62. (**C**) Representative VLP speed and intensity graphs (top) and kymographs (merged, M-mCherry, and M-pHluorin) in all dimensions (bottom) for a single VLP^Omi^:M^Ch^M^pH^R undergoing internalization in an A549 cell. In the example, the speed increases in parallel with pHluorin signal decrease. (**D**) Same as (**C**) but for VLP^del-1^:M^Ch^M^pH^R. (**E**) Distribution of time intervals between VLP binding and start of pHluorin intensity decrease for individual VLP^Wu^:M^Ch^M^pH^R, VLP^Omi^:M^Ch^M^pH^R, and VLP^del-1^: M^Ch^M^pH^R during internalization in A549 cells. Two-tailed Student’s *t*-test; NS *p* > 0.01. VLP^Wu^:M^Ch^M^pH^R n = 55, for VLP^Omi^:M^Ch^M^pH^R n = 48, for VLP^del-1^:M^Ch^M^pH^R n = 62. (**F**) Distribution of time intervals between VLP binding and start of speed increase for individual VLP^Wu^:M^Ch^M^pH^R, VLP^Omi^:M^Ch^M^pH^R, and VLP^del-1^:M^Ch^M^pH^R during internalization in A549 cells. Two-tailed Student’s *t*-test; NS *p* > 0.01. For A549-WT n = 55, for A549-Omi n = 48, for A549-del1 n = 62. (**G**) Distribution of time intervals between VLP intensity decrease and start of speed increase for individual VLP^Wu^:M^Ch^M^pH^R, VLP^Omi^:M^Ch^M^pH^R, and VLP^del-1^:M^Ch^M^pH^R during internalization in A549 cells. Two-tailed Student’s *t*-test; NS *p* > 0.01. For VLP^Wu^:M^Ch^M^pH^R n = 55, for VLP^Omi^:M^Ch^M^pH^R n = 48, for VLP^del-1^:M^Ch^M^pH^R n = 62.

In contrast to SARS-CoV, SARS-CoV-2 harbors a PRRA furin cleavage site (FCS) at the S1/S2 junction of the S protein [53]. Cleavage by cellular protease furin followed cleavage at the S2′ site by TMPRSS2 or cathepsins results in the separation of the two S sub-domains. Thus, we sought to determine how absence of the FCS would affect internalization at the single VLP level. To this end, we used VLPs containing M-mCherry, M-pHluorin, and S protein lacking an FCS (del-1) [54,55,56,57,58]. We refer to these as VLP^Wu(del-1)^:M^Ch^M^pH^,R, short for VLP^Wuhan (del-1)^:(N, E, S, M&M-mCherry & M-pHluorin, T20 RNA). Treatment of A549 cells overexpressing ACE2 and TMPRSS2 with VLP^Wu(del-1)^:M^Ch^M^pH^,R, revealed a decrease in the percentage of VLPs for which pHluorin signal decrease occurred without a change in mCherry intensity, from 89% for VLP^Wu^:M^Ch^M^pH^, R to 73% for VLP^Wu(del-1)^:M^Ch^M^pH^, R (Figure 4A). Further, the rate of pHluorin decrease was similar between the two (Figure 4B). The distribution of the time intervals between VLP binding and pHluorin decrease/VLP speed increase was also comparable (Figure 4C–G, Table 1). As observed for VLP^Wu^, the pH decrease of VLP^Wu(del-1)^ initiated either a little before, in parallel to, or after the speed of the same VLPs began to increase (Figure S7; Videos S22 and S23). Similar results were obtained in VeroE6 cells (Figures S8 and S9; Videos S24 and S25). Taken together, our results indicate no significant role for the FCS in the internalization of SARS-CoV-2 VLPs.

### 3.5. Kinetics of Omicron VLPs

Emergence of the SARS-CoV-2 Omicron variant in the autumn of 2021 was followed by its rapid spread, overtaking previous variants in global prevalence. Omicron harbors more than 50 amino acid substitutions, 37 of which in the S protein, with 15 affecting the receptor-binding domain. In light of its considerable transmissibility and rapid replication in human bronchi (70-fold greater than of previous variants), we sought to measure the internalization kinetics of Omicron [59]. To this end, we employed VLPs composed of unlabeled N, E, S, and M proteins harboring Omicron substitutions. VLPs also included mCherry-tagged and pHluorin-tagged M protein and are referred to as VLP^Omi^:M^Ch^M^pH^R, short for VLP^Omicron^:(N, E, S, M, M-mCherry & M-pHluorin, T20 RNA). Treatment of A549 cells overexpressing ACE2 and TMPRSS2 (Videos S26–S28) revealed a decrease in the proportion of VLPs in which the pHluorin signal decay occurred without a change in mCherry signal, that is, from 89% in VLP^Wu^:M^Ch^M^pH^R to 65% for VLP^Omi^:M^Ch^M^pH^R (Figure 4A). However, the rate of decrease pHluorin intensity was largely identical between the two VLP types as well as the Wuhan del-1 mutant. The average and the distribution of intervals between VLP binding and pHluorin decrease/VLP speed increase were also comparable among the three VLP types (Figure 4E and Figure S10). Taken together, no considerable differences in internalization kinetics were noted for the Omicron variant.

### 3.6. Rate of VLP Nucleocapsid Release

**Figure 5:**
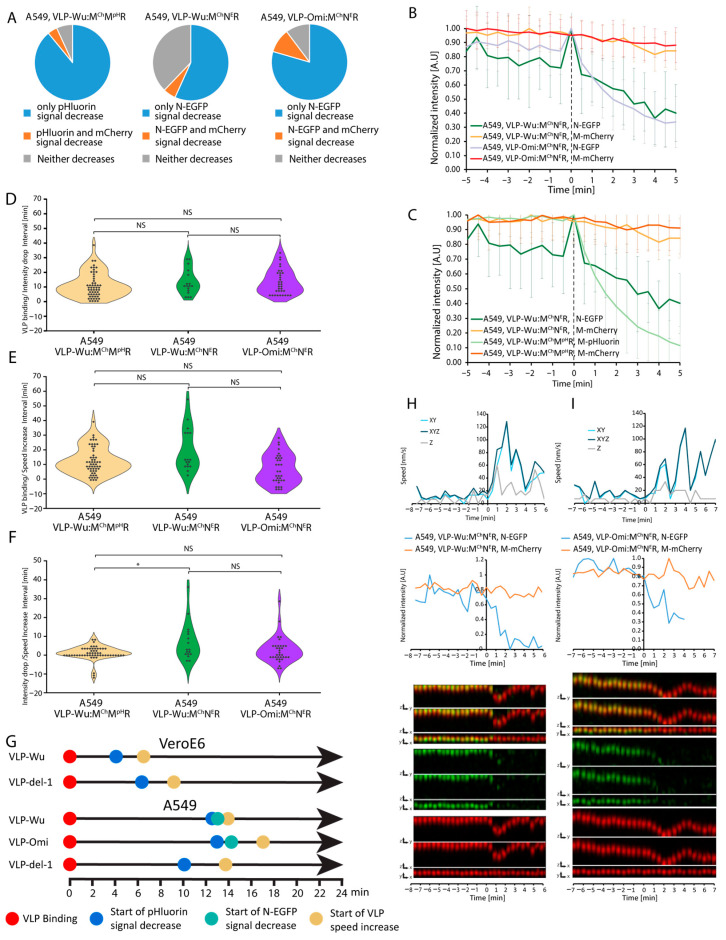
Comparison of VLP binding, acidification, and speed increase dynamics between VLP^Omi^:M^Ch^N^E^R, VLP^Wu^:M^Ch^N^E^R, and VLP^Wu^:M^Ch^M^pH^R during internalization in A549 cells. (**A**) Percentage of VLPs in which only the signal intensity of N-EGFP (middle and right) or M-pHluorin (left) decreases (blue), the intensities of both N-EGFP/M-pHluorin and M-mCherry decrease simultaneously (orange), and neither decreases (gray). VLPs were tracked for 100 min after addition. (**B**) Comparison of N-EGFP intensity decrease rate during VLP^Omi^:M^Ch^N^E^ R and VLP^Wu^:M^Ch^N^E^R internalization in A549 cells. The average intensity of N-EGFP is presented as a function of time where the individual VLPs were aligned to the start of N-EGFP signal decrease (0 min). The average M-mCherry intensity is also presented. Error bars represent the standard deviation. For A549 VLP^Wu^:M^Ch^N^E^R n = 17, for A549 VLP^Omi^:M^Ch^N^E^ n = 34. (**C**) Comparison of M-pHluorin and N-EGFP intensity decrease rates during internalization of VLP^Wu^:M^Ch^M^pH^R and VLP^Wu^:M^Ch^N^E^R, respectively, in A549 cells. The average intensities of N-EGFP and M-pHluorin are presented as a function of time where the individual VLPs were aligned to the start of VLP N-EGFP or M-pHluorin decrease (0 min). The average M-mCherry intensity of the same particles is also presented. Error bars represent the standard deviation. For A549 VLP^Wu^:M^Ch^M^pH^R n = 55, for A549 VLP^Wu^:M^Ch^N^E^R n = 17. (**D**) Distribution of time intervals between VLP binding and the start of pHluorin/N-EGFP intensity decrease for individual VLP^Wu^:M^Ch^M^pH^R, VLP^Wu^:M^Ch^N^E^R, and VLP^Omi^:M^Ch^N^E^R during internalization in A549 cells. Two-tailed Student’s *t*-test; NS *p* > 0.01. For A549 VLP^Wu^:M^Ch^N^E^R n = 17, for VLP^Omi^:M^Ch^N^E^R n = 34, and for VLP^Wu^:M^Ch^M^pH^R n = 55. (**E**) Distribution of time intervals between VLP binding and start of speed increase for individual VLP^Wu^:M^Ch^M^pH^R, VLP^Wu^:M^Ch^N^E^R, and VLP^Omi^:M^Ch^N^E^R during internalization in A549 cells. Two-tailed Student’s *t*-test; NS *p* > 0.01. For A549 VLP^Wu^:M^Ch^N^E^R n = 17, for A549 VLP^Omi^:M^Ch^N^E^R n = 34, and for A549 VLP^Wu^:M^Ch^M^pH^R n = 55. (**F**) Distribution of time intervals between start of pHluorin/N-EGFP intensity decrease and start of speed increase for individual VLP^Wu^:M^Ch^M^pH^R, VLP^Wu^:M^Ch^N^E^R, and VLP^Omi^:M^Ch^N^E^R during internalization in A549 cells. Two-tailed Student’s *t*-test; NS *p* > 0.01; * *p* < 0.01. For A549 VLP^Wu^:M^Ch^N^E^R n = 17, for A549 VLP^Omi^:M^Ch^N^E^R n = 34, and for A549 VLP^Wu^:M^Ch^M^pH^R n = 55. (**G**) Schematic of the major internalization-associated events through time for wild-type and mutant VLPs in VeroE6 and A549 cells. (**H**) Representative VLP speed and intensity graphs (top) and kymographs (merged, M-mCherry, and N-EGFP) in all dimensions (bottom) for a single VLP^Wu^:M^Ch^N^E^R undergoing internalization in an A549 cell. In this example, the speed increases in parallel with pHluorin signal decrease. (**I**) Same as (**H**) for VLP^Omi^:M^Ch^N^E^R.

**Figure 6:**
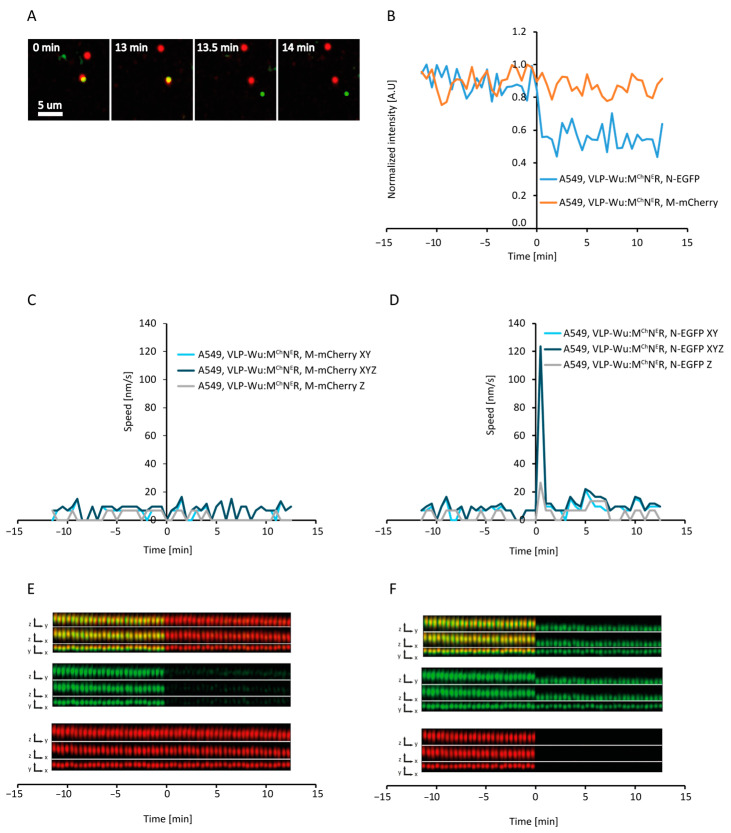
Tracking of nucleocapsid release following VLP internalization. (**A**) Time-lapse images of N-EGFP release during internalization of VLP^Wu^:M^Ch^N^E^R in A549 cells where the separation of the nucleocapsid (N-EGFP) from the VLP membrane (M-mCherry) can be observed. (**B**) Changes in N-EGFP and M-mCherry during nucleocapsid release for the above VLP (tracked based on the N-EGFP signal). (**C**) VLP speed profile during nucleocapsid release measured based on M-mCherry movement for the same VLP. (**D**) VLP speed profile during nucleocapsid release measured based on N-EGFP movement for the same VLP. Note the speed increase during nucleocapsid release, which is missing in (**C**). (**E**) Kymogram in all dimensions measured based on M-mCherry tracking. (**F**) Kymogram in all dimensions based on N-EGFP tracking. Note the change in movement along the Z-axis during nucleocapsid release, which is missing in (**E**).

Nucleocapsid release occurs via VLP fusion either to the plasma or endosomal membrane, representing an essential step in the internalization process. Thus, we set out to measure the dynamics of VLP nucleocapsid release, using VLPs in which a fraction of the nucleocapsid-forming N protein is tagged with EGFP, and a fraction of M is tagged with mCherry (VLP^Wu^:N^eG^M^Ch^R), short for SARS-CoV-2:(E, S, N&N-eGFP, M&M-mCherry, T20 RNA) VLPs. We observed that, while initially greater than 90% of VLPs emitted both green and red fluorescence, less than 1% of VLPs emitted green fluorescence at 2 days after production. Although we have previously shown that fluorescently tagging M or N with GF results in stable VLPs as imaged by atomic force microscopy [12]), our current observations suggest that tagging both M and N proteins simultaneously reduces VLP stability [13]. In 57% of these double-labeled VLPs, the EGFP signal disappeared before the mCherry one, which may reflect N protein release (Figure 5A). The rate of N-EGFP signal decay in VLP^Wu^:N^eG^M^Ch^R was slower than that of M-pHluorin intensity decrease in VLP^Wu^:M^Ch^M^pH^R (Figure 5C). The observed slow decay suggests that nucleocapsid release may not be a single-step rapid event, but a rather gradual process of continuous N-EGFP release, which cannot be followed thereafter. Only in a single case (of 18 VLPs) were we able to follow the N-eGFP signal after rapid release of the nucleocapsid. We tracked both fluorescent signals (M-mCherry and N-eGFP) for this VLP and observed that the M-mCherry signal did not move in z during signal separation, while the eGFP did. This could suggest that, in this case, fusion occurs either at the cell plasma membrane or during VLP ingression, prior to active movement via the microtubular network (Figure 6 and Video S29). On average, there was no statistically significant difference between the interval from VLP binding to the start of pH decrease of VLP^Wu^:M^Ch^M^pH^R (Table 1) and the interval from VLP binding to the start of nucleocapsid release of VLP^Wu^:N^eG^M^Ch^R (Figure 5D–F). This result suggests that, on average, VLP acidification coincides with nucleocapsid release.

Next, we sought to determine nucleocapsid release kinetics in Omicron VLP^Omi^:M^Ch^N^eE^R, short for SARS-CoV-2^Omicron^:(E, S, M&M-mCherry, N&N-eGFP, T20 RNA) VLPs. Surprisingly, the percentage of intact, double-labeled particles was considerably higher among VLP^Omi^:M^Ch^N^eE^R (2–3%) as opposed to their VLP^Wu^:M^Ch^N^eE^R counterparts (<1%), while remaining much lower than that among VLP^Omi^:M^Ch^M^pH^R (>90%). In approximately 80% of VLP^Omi^:M^Ch^N^eE^R, N-eGFP fluorescence disappeared before mCherry, reflecting a 23% more effective release than observed for VLP^Wu^:M^Ch^N^eE^R (Figure 5A). However, the rates of N-eGFP signal decrease (Figure 5B) were similar between the Wuhan and Omicron VLPs, which suggests that nucleocapsid release occurred at a comparable rate. In addition, there was no statistically significant difference between the interval from VLP binding to nucleocapsid release for the Wuhan and Omicron VLPs (Figure 5D, Table 1). Thus, Omicron mutations had no effect on the nucleocapsid release step of internalization. In the Omicron VLP, speed increase occurred shortly after nucleocapsid release, as observed for Wuhan VLP (Figure 5E,F, Table 1) indicating that VLP membrane fusion occurs, on average, before the start of microtubule-mediated transport. However, at the single-VLP level, we observed cases of speed increase occurring after, in parallel to, or before nucleocapsid release (Figure 5H,I, Figures S11 and S12, and Videos S30–S33), indicating no causality between these two events, as was also observed above for the pH decrease.

## 4. Discussion

In this work, we developed a customized software pipeline (SPARTACUSS) to reconstruct the timeline of SARS-CoV-2 internalization through comprehensive kinetic characterization of five key events during viral internalization, at the single-VLP level—VLP binding, start of pH decrease, start of nucleocapsid release, dynamin-VLP co-localization, and start of active microtubule-dependent VLP movement.

Comparison between the timing of these events demonstrates that about 4 min after VLP binding to Vero E6 cells and 12 min after VLP binding to A549 cells, pH starts to rapidly decrease, which coincides with dynamin binding and nucleocapsid release, quickly followed by the initiation of active microtubule-dependent VLP motion. Our results suggest that VLP fusion (N-nucleocapsid release) occurs simultaneously with or shortly after endosome formation. Surprisingly, the VLPs do not co-localize with Rab-5a-positive early endosomes during VLP internalization. The two to three times shorter time required for VLP internalization in VeroE6 cells compared to A549 cells could explain VeroE6 higher susceptibility to SARS-CoV-2 infection. We also evaluated the effects of ACE2 and TMPRSS2 overexpression on VLP internalization dynamics and efficiency. Interestingly, while we observed a considerable increase in VLP uptake efficiency (i.e., a greater proportion of VLPs were internalized), there was no effect on the dynamics of specific steps in the internalization process, namely, pH decrease and speed increase. We consider it plausible that an abundance of ACE2 and TMPRSS2 would facilitate greater particle binding on the cell membrane, accounting for the highly effective internalization observed. However, once bound to the host cell membrane, the subsequent steps occur with similar kinetics, as the overexpressed proteins do not affect the events downstream of membrane binding (e.g., endocytosis and related events, such as the pH decrease and speed increase via microtubule transport). It should be noted that an increasing number of factors beyond the well-established ACE2 and TMPRSS2 are shown to affect uptake/internalization efficiency, including heparan-sulfate proteoglycans and syndecans. Their relevance to internalization dynamics, however, is unclear and thus remains to be addressed in future research [60,61].

In the present work, neither Omicron or del-1 mutations influence the internalization steps of SARS-CoV-2 VLPs. It is known that passage of SARS-CoV-2 in VeroE6 cells leads to mutations in the S1/S2 junction, suggesting that the FCS is dispensable for virus propagation, in line with our results [58,62]. The lack of significant difference in internalization dynamics between VLPs with and without FCS deletion observed herein may be attributed to non-effective cleavage by furin during maturation even in VLPs with an intact FC. Our Omicron data suggest that the considerable number of S mutations and the greater transmissibility observed in humans are not related to pronounced differences in internalization kinetics. Thus, these results point away from enhanced internalization as a basis for superior transmissibility, suggesting greater stability or replication efficiency as potential underlying mechanisms.

It is our vision that the comprehensive measurement of the SARS-CoV-2 VLP internalization steps can be utilized in the evaluation of novel antiviral therapeutics, in addition to shedding novel insights into the molecular mechanisms driving the viral life cycle.

## 5. Conclusions

In summary, the current work provides a novel live-cell imaging-based pipeline for studying VLP internalization kinetics at a single-VLP level. Utilizing this approach, we meticulously characterize the timing of key events during the internalization process in two widely used SARS-CoV-2 infection model cell lines. Furthermore, we compared whether the differences in infectivity observed for the del-1 and Omicron variants can be attributed to differences in their internalization dynamics.

## Figures and Tables

**Table 1:** Measured intervals between different steps of VLP internalization.

VLP Variants/Cell Line	VLP Binding to pH Drop or N Release	pH Drop or N Release to Speed Increase	VLP Binding to Speed Increase
VLP-WT MM in Vero E6	4.1 ± 3.6 min	2.4 ± 3.7 min	6.5 ± 5.4 min
VLP-WT MM in A549	12.5 ± 8.4 min	1.4 ± 3.7 min	13.9 ± 9.2 min
VLP-WT NM in A549	13 ± 8.6 min	7.0 ± 10.0 min	20.1 ± 14.8 min
VLP-Omi NM in A549	14.2 ± 8.5 min	2.9 ± 6.9 min	17.1 ± 10.1 min
VLP-del1 MM in Vero E6	6.4 ± 5.4 min	2.8 ± 8.2 min	9.2 ± 8.9 min
VLP-del1 MM in A549	16.7 ± 12.7 min	1.7 ± 1.6 min	18.4 ± 12.9 min
VLP-del1 MM in A549	10.12 ± 8.72 min	3.61 ± 7.79 min	13.74 ± 11.57 min
VLP-OMI MM in A549	13.01 ± 8.4 min	4.03 ± 7.44 min	17.04 ± 11.48 min

## Data Availability

To make the results publicly available we generated a database with representative videos for all the experiments at COVIDynamics.imb.bas.bg. They will be searchable and accessible. The raw data will be provided upon reasonable request. The software will be open-access with a detailed, step-wise manual and everybody will be able to use it.

## Supplementary Materials

The following supporting information can be downloaded at: https://www.mdpi.com/article/10.3390/v16081341/s1, Figure S1: Internalization of a SARS-CoV-2Wu:(E,S,M) observed via thin-section electron microscopy of murine lung epithelial (*MLE*) *cell*s. Figure S2: Schematic representation of the SPARTACUSS pipeline for measurement and analysis of VLP internalization dynamics. Figure S3: Example of single VLP^Wu^:M^Ch^M^pH^R entries into A549 cells. Figure S4: Example of single VLP^Wu^:M^Ch^M^pH^R entries into Vero E6 cells. Figure S5: Comparison of VLP^Wu^:M^Ch^M^pH^R binding, acidification, and speed increase dynamics in A549 cells with and without overexpression of ACE2 and TMPRSS2. Figure S6: Comparison of VLP^Wu^:M^Ch^M^pH^R binding, acidification, and speed increase dynamics in VeroE6 cells with and without overexpression of ACE2 and TMPRSS2. Figure S7: Example of single VLP^del-1^:M^Ch^M^pH^R entry into A549 cells. Figure S8: Dynamics of VLP binding and speed increase for VLP^Wu^:M^Ch^M^pH^ and VLP^del-1^:M^Ch^M^pH^ during internalization in VeroE6 cells. Figure S9: Examples of a single VLP^del-1^:M^Ch^M^pH^ entry into Vero E6 cells. Figure S10: Examples of single VLP^Omi^:M^Ch^M^pH^R entries into A549 cells. Figure S11: Examples of single VLP^Omi^:M^Ch^N^E^R entries into A549 cells. Figure S12: Examples of single VLP^Wu^:M^Ch^N^E^R entries into A549 cells. Video S1: U2OS ACE2 Neon Green No Tracks. Video S2: U2OS ACE2 Neon Green with 2 Tracks. Video S3: U2OS ACE2 Neon Green with many Tracks. Video S4: U2OS mNeon Green No Tracks. Video S5: U2OS mNeon Green One Track. Video S6: U2OS Neon Green many Tracks. Video S7: Combined Video from mNeon Green original transparent and volumized. Video S8: Tubulin. Video S9: Anti SARS-CoV-2 AB. Video S10: Dynamin-1-GFP track15 slowed down. Video S11: Dynamin-1-GFP with many tracks. Video S12: Dynamin-1-GFP with one track. Video S13: Vero E6 Dynol 34-2. Video S14: early endosomes. Video S15: Acidic Vesicles. Video S16: Vero with M-pHLuorin. Video S17: A549 with M-pHLuorin. Video S18: WT Vero MM With No Tracks. Video S19: WT Vero MM With many Tracks. Video S20: WT A549 MM no tracks. Video S21: WT A549 MM with tracks. Video S22: Del-1 A549 no tracks. Video S23: Del-1 A549 with tracks. Video S24: Del-1 Vero no tracks. Video S25: Del-1 Vero with tracks. Video S26: Omi MM no tracks. Video S27: Omi MM one track. Video S28: Omi MM many tracks. Video S29: Particle Splitting. Video S30: WT A549 NM no tracks. Video S31: WT A549 NM with tracks. Video S32: Omi A549 NM no tracks. Video S33: Omi A549 NM with tracks. Software-SPASRTACUSS.zip.
